# Sedentary behaviour among older adults residing in flat and hilly neighbourhoods and its association with frailty and chronic disease status

**DOI:** 10.1186/s12889-023-17029-0

**Published:** 2023-10-24

**Authors:** Nestor Asiamah, Simon Mawulorm Agyemang, Edgar Ramos Vieira, Hafiz T. A. Khan, Janvier Gasana

**Affiliations:** 1 School of Health and Social Care, Division of Interdisciplinary Research and Practice, University of Essex, Colchester, Essex CO4 3SQ UK; 2 Department of Science/Health, Physical Education and Sports, Abetifi Presbyterian College of Education, P.O. Box 19, Abetifi – Kwahu, E/R Ghana; 3 Department of Physical Therapy, Nicole Wertheim College of Nursing & Health Sciences, Florida International University, Miami, FL USA; 4 College of Nursing, Midwifery, and Healthcare, University of West London, Paragon House, Boston Manor Road, Brentford, TW8 9GB UK; 5 Department of Community Health, Kuwait University, Kuwait City, Kuwait

**Keywords:** Ageing, Sedentary behaviour, Older adults, Frailty, Poor health, Chronic disease status, Ghana

## Abstract

**Background:**

Living in hilly neighbourhoods can be associated with sedentary behaviour, but no study has compared sedentary behaviour and its associations with frailty, chronic diseases, and poor health between flat and hilly neighbourhoods among older adults. This study, therefore, compared older adults’ sedentary behaviour and its association with frailty, poor health, and chronic disease status between low and hilly neighbourhoods.

**Methods:**

This study utilised a STROBE-compliant cross-sectional design with sensitivity analyses and a common methods bias assessment. The participants were 1,209 people aged 50^+^ years who resided in flat (Ablekuma North, *n* = 704) and hilly (Kwahu East, *n* = 505) neighbourhoods in Ghana. The data were analysed with the independent samples *t*-test and hierarchical linear regression.

**Results:**

Older adults in the hilly neighbourhood were more sedentary than those in the flat neighbourhood. The association between sedentary behaviour and chronic disease status was significant in both neighbourhoods, but this relationship was stronger in the hilly neighbourhood. Older adults in the flat neighbourhood reported lower sedentary behaviour at higher frailty (β = -0.18; t = -3.2, *p* < 0.001), but those in the hilly neighbourhood reported higher sedentary behaviour at higher frailty (β = 0.16; t = 3.54, *p* < 0.001).

**Conclusions:**

Older adults living in the hilly neighbourhood reported higher sedentary behaviour. In the hilly neighbourhood, sedentary behaviour was more strongly associated with frailty and chronic disease status. Older adults in hilly neighbourhoods may need extra support to avoid sedentary behaviour.

**Supplementary Information:**

The online version contains supplementary material available at 10.1186/s12889-023-17029-0.

## Introduction

A hallmark for ageing in optimal health is maintaining Physical Activity (PA) and avoiding sedentary behaviour, both of which have been evidenced to protect against chronic conditions [1–3] and early mortality [1, 2]. Sedentary behaviours are awake periods with energy expenditure ≤ 1.5 basal metabolic rate [4]. Interventions enabling individuals to avoid sedentary behaviour should be a top public health agenda. Understanding factors that influence sedentary behaviour is necessary for developing and rolling out these interventions.

A factor influencing sedentary behaviour is whether the neighbourhood is steeply hilly or not [5]. Several studies [5–7] have shown that residents living in hilly neighbourhoods are less likely to perform PA. In a qualitative study undertaken in Sweden [6], for example, older adults revealed that they did not perform PA because of hills within their neighbourhoods. Older adults are generally frail and may lack the functional ability for climbing and descending hills. Moving up and down steep hills is analogous to using stairs, which can be associated with falls in older adults [8]. Older adults would avoid types of PA (e.g., jogging or walking in a hilly neighbourhood) that their physical functional ability cannot support. PA performed indoors has become a popular option [9], but this alone may not sufficiently buffer sedentary behaviour. If so, older adults living in hilly neighbourhoods, compared with those living in flatter neighbourhoods, can be expected to report higher sedentary behaviour.

The hilly neighbourhood considered in this study is characterised by steep and rocky hills, and may seem unsafe for active forms of transportation (e.g., walking, bicycling, and skating) by which residents often exercise in their communities [10, 11]. Older adults living in such a neighbourhood may find it more difficult to perform PA and avoid sedentary behaviour. The flat neighbourhood without hills would require less energy expenditure in PA. Contrary to the above evidence, nevertheless, a few studies [12, 13] have reported that living in hilly neighbourhoods can be associated with higher PA. The mixed evidence necessitated the current study and calls for a better understanding of potential differences in sedentary behaviour between the two neighbourhoods.

Whether older adults living in hilly neighbourhoods would perform PA and avoid sedentary behaviour would depend on their perceived health. For example, older adults who rate their health as poor are likely to feel unsafe performing PA in hilly neighbourhoods. This feeling may stem from underlying chronic conditions such as arthritis, osteoporosis, and frailty, all of which are more prevalent in older populations [14, 15]. Thus, three indicators of health [i.e., frailty, chronic disease status, and poor health] may more strongly predict sedentary behaviour in hilly neighbourhoods. Since these health problems grow stronger over the life course [16, 17], their associations with sedentary behaviour may depend on age, especially in hilly neighbourhoods. This study, therefore, aimed to compare sedentary behaviour and its associations with the above health indicators between low and hilly neighbourhoods, with covariates including age adjusted for.

This study was undertaken in response to a review [4] calling for studies assessing sedentary behaviour and its correlates in multiple contexts (e.g., neighbourhoods with inequalities and barriers to PA). By utilising a clinical measure of frailty, this study reports implications for geriatric care and clinical practice. This study is also expected to guide individuals to choose their neighbourhoods or retirement villages in later life. Apart from being the first to compare sedentary behaviour and its associations with the health indicators between low and hilly neighbourhoods, it employed a robust cross-sectional design that may serve as a model for future research.

## Methods

### Design

This study utilised a cross-sectional design compliant with the STROBE (i.e., Strengthening the Reporting of Observational Studies in Epidemiology). This design included a common methods bias (CMB) assessment and sensitivity analyses performed with a hierarchical linear regression (HLR) analysis.

### Neighbourhoods and samples

The study participants were community-dwelling older adults aged 50 + years residing in the flat (located in Ablekuma North District, Accra) and hilly (located in Abetifi, Kwahu East District) neighbourhoods. Table 1 shows relevant attributes of both neighbourhoods, including an elevation level of 116 m for the flat neighbourhood and 601 m for the hilly neighbourhood. Google Maps was used to confirm the elevations of the two neighbourhoods. As the summary statistics reported in this study later indicate, the two samples were associated with similar age distributions.

**Table 1:** Attributes of the districts in which the flat and hilly neighbourhoods were located

Attribute	Area	
	Flat neighbourhood (Ablekuma North District)	Hilly neighbourhood (Kwahu East District)
Elevation (above sea level)	116 m	601 m
Greenness	Mostly not green	Mostly green
Flatness of streets	Mostly levelled and tarred streets	Mostly untarred and hilly streets
Connectivity of streets	Mostly connected	Mostly unconnected due to hills
Mixed land use	Higher	Low
Population density	14,000/km^2^	824/km^2^
Neighbourhood type	Urban	Semi-urban
Region	Greater Accra	Eastern Region
Population	159,208	79,726

### Participant selection

The following selection criteria were used to select the participants: (1) being aged 50 years or higher, (2) having a minimum of a basic education qualification, which evidenced participants’ ability to complete questionnaires in English, (3) not having any health problem or physiological limitations that precluded PA [18], and (4) willingness to participate in the study voluntarily. Individuals were screened against these criteria through a structured interview lasting between 5 to 10 min. The G*Power software and recommended statistics (i.e., effect size = 0.2, α = 0.05; power = 0.8) [19] were used to calculate the minimum sample size necessary. The minimum sample reached for using HLR with a maximum of 9 predictors was 88. There was no sampling frame for this study, so non-probabilistic sampling (i.e., convenience sampling) was adopted to select the participants. The participants were selected at community centres and social events (e.g., conferences, seminars, and church activities) that included potential participants of this study. A total of 1292 eligible individuals (i.e., flat neighbourhood = 741; hilly neighbourhood = 551) were selected and interviewed. We gathered data on all 1292 eligible individuals to maximise the power of our tests.

### Measures

We adopted a method from previous research to measure sedentary behaviour as time [in minutes] spent on a typical day sitting in different situations [20]. Additional file 1 shows items used to measure sedentary behaviour. Frailty was measured with the standardised 15-item Tilburg Frailty Indicator with a dichotomous descriptive anchor (i.e., no – 1, and yes – 2) that was adopted in whole from a previous study [21]. This scale is a tool used in clinical practice to measure frailty as general weakness of the body and the condition of being delicate. It was used because it is the most widely used measure of frailty in a clinical context; measuring frailty in a clinical context was the ideal way to identify implications for clinical practice. The scale produced a satisfactory Cronbach’s α ≥ 0.7 (flat neighbourhood = 0.83, and hilly neighbourhood = 0.79) in the current study. Data on frailty were generated by summing up scores from its items. Additional file 2 shows items of the scale used to measure frailty.

Chronic disease status was measured following previous research [18] by asking participants to report the number of chronic conditions they had. The resulting data were split into two categories (i.e., none – 1, and one or more– 2), coded into a dummy-type variable, and “none” made a reference category in the analysis. Poor health was measured by asking the participants to rate their health (i.e., poor health – 1, and good health – 2) [22], coding the resulting variable into a dummy-type variable, and making “good health” a reference.

Potential covariates (i.e., gender, relationship status, income, education, and age) that were previously reported to be associated with health indicators and sedentary behaviour were measured and included in the analysis [20, 23–26]. Gender (men – 1, and women – 2) and relationship status (“not in a relationship” – 1, and “in a relationship” – 2) were measured as categorical variables and coded into dummy-type variables for HLR analysis. We operationalised “relationship status” by asking the participants to indicate whether they were married or lived with a partner. Income was measured as a discrete variable by asking the participants to report their net monthly income in Ghana cedis. Age was measured as a discrete variable (in years) by asking the participants to report their age. Education was a discrete variable measured as the individual’s years of schooling.

### The questionnaire

A self-reported questionnaire with two main sections was utilised to collect the data. The first section presented items on frailty whereas the second section captured questions measuring poor health, chronic disease status, and covariates. The questionnaire had an introductory section that presented the study aim, inclusion criteria, ethical statement, and instructions for respondents. We followed procedures recently applied [22, 27] to minimise or avoid CMB. First, we provided background information that enabled respondents to understand the context in which each variable was measured. Measures were put in distinct sections or subsections with the relevant instructions, enabling the participants to respond uniquely to each measure. Finally, we employed the one-factor method [22, 28] to assess CMB. Regarding this method, we employed exploratory factor analysis with varimax rotation to assess the factor structure of the frailty scale used. This technique produced a satisfactory factor solution with not less than three factors (i.e., flat neighbourhood: total variance = 59.9%, number of factors = 3; factor loading ≥ 0.5, and variance of the first factor = 29.3%; hilly neighbourhood: total variance = 64.2%, number of factors = 6; factor loadings ≥ 0.5; variance of the first factor = 20.9%). The largest variances produced were less than 40% as recommended [27, 28]. Thus, CMB was avoided or minimised.

### Data collection

This study received ethics review and approval from the ethics review board of the Africa Centre for Epidemiology in Accra (# 005-10-2022-ACE). All research protocols were approved by the above ethics review committee, and the participants provided written informed consent before participating in this study. All methods and procedures were carried out in accordance with ethical regulations and guidelines such as the Declaration of Helsinki. The questionnaires were hand-delivered to the participants by research assistants at the community centres where they were recruited. Data were gathered over about six weeks (20th December 2022 to 8th January 2023). Out of 1292 questionnaires administered, 83 (i.e., flat neighbourhood = 37, and hilly neighbourhood = 46) were discarded because they were completed halfway or were not completed at all. So, 1209 questionnaires (i.e., flat neighbourhood = 704, and hilly neighbourhood = 505) were analysed.

### Statistical analysis method

The data were analysed with the SPSS version 28 (IBM New York, USA) in two main phases. The first phase was the exploratory stage where the data were summarised, missing items and outliers identified, key assumptions assessed, and the first sensitivity analysis performed. We summarised the data with descriptive statistics; continuous and discrete variables were summarised with the mean whereas categorical variables were summarised with frequencies. The data contained less than 10% of missing items, so we analysed the data without removing the missing data following previous research [22]. To identify outliers, we followed previous procedures for assessing the normal distribution of the data [22, 29]. The other assumptions assessed for using the independent samples t-test and HLR analysis were (1) homogeneity of variances of the samples; (2) linearity of the tested associations, (3) independence of errors, (4) homogeneity of error variances around the regression line, and (5) multicollinearity among the predictors. Additional file 3 shows the steps taken to assess and meet these assumptions for both samples.

We performed the first sensitivity analysis to identify the ultimate covariates following a statistical technique previously utilised [22]. This analysis enabled us to assess the respective influences of the covariates (e.g., age) on the primary associations tested and to remove variables that were unlikely to confound the primary associations from the analyses. It assumes that not all potential covariates can affect the relationships being tested [30, 31]. Additional file 4 shows the steps taken in this analysis. Age, gender, and income were selected in this analysis as the ultimate covariates and incorporated into the final regression models.

**Fig. 1:**
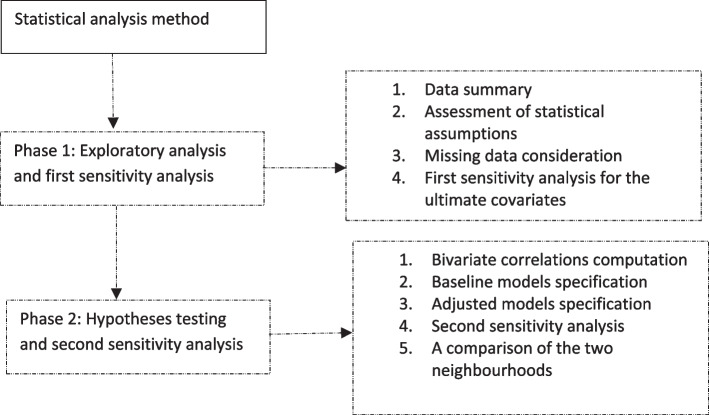
A flow chart of the statistical analysis strategy

**Fig. 2:**
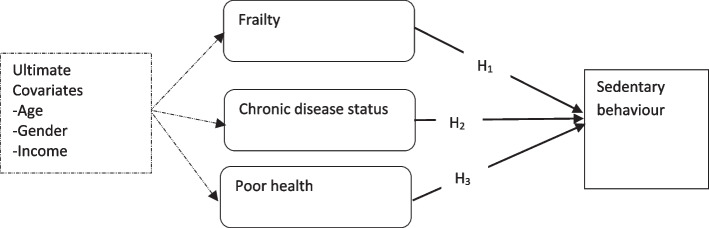
The associations of sedentary behaviour with frailty, chronic disease status, and poor health. Note: Broken arrows represent the influence of covariates; H_1_-H_3_ are hypotheses or associations compared between the two neighbourhoods; H_1_ – Frailty is associated with sedentary behaviour; H_2_ – chronic disease status (i.e., having one or more chronic conditions) is associated with sedentary behaviour, and H_3_ – poor (self-reported) health is associated with sedentary behaviour

The second phase of the analysis aimed to compare sedentary behaviour and its associations with the health indicators between the two neighbourhoods. Figure 1 shows the associations or hypotheses tested. We compared sedentary behaviour between two neighbourhoods with the independent samples *t*-test. To test the three hypotheses, we first assessed bivariate correlations between the variables using Pearson’s correlation coefficients. We then fitted three regression models. The first model was a non-adjusted or baseline model that assessed the associations of the three health indicators with sedentary behaviour for the flat neighbourhood. The second model (i.e., the age-adjusted model) adjusted for only age by infusing age into the first baseline model. We included this model to demonstrate the unique influence of age on the primary associations. The final model is the ultimate model infusing all the ultimate covariates. Hence, this study’s conclusions were based on it. We fitted three similar models for the hilly neighbourhood. Following previous research [22], we performed a second sensitivity analysis by comparing the standardised regression weights of the baseline model to the coefficients of the age-adjusted and ultimate models. We detected the statistical significance of each test at a minimum of *p* < 0.05. Figure 2 is a flow chart of the statistical analysis strategy utilised.

## Findings

In Table 2, 52% (*n* = 368) of the participants were men in the flat neighbourhood whereas 44% (*n* = 220) were men in the hilly neighbourhood. Average ages in the flat and hilly neighbourhoods were respectively about 63 years (Mean = 62.91; SD = 9.29) and 61 years (Mean = 61.31; SD = 9.95). Thus, the two samples had similar age distributions characterised by nearly equal means and standard deviations. The average sedentary behaviour was about 89 min (Mean = 88.74; SD = 43.8) for the flat neighbourhood and 1032 min (Mean = 1032.36; SD = 1086.78) for the hilly neighbourhood. The sedentary behaviour reported by participants in the hilly neighbourhood was significantly larger (t = -18.10; *p* < 0.001; Cohen’s d effect size = 673.322); sedentary behaviour in the hilly neighbourhood was 1063% larger.

**Table 2:** Summary statistics on study variables between flat and hilly neighbourhoods

Variable	Category	Flat neighbourhood (*n* = 704)		Hilly neighbourhood (*n* = 505)	
		n/Mean	%/SD	n/Mean	%/SD
Categorical variables					
Gender	Men	368	52.27	220	43.56
	Women	332	47.16	285	56.44
	Missing	4	0.57	0	0
	Total	704	100	505	100
Chronic disease status	None	187	26.56	250	49.5
	One or more	517	73.44	250	49.5
	Missing	0	0	5	0.99
	Total	704	100	505	100
Relationship status	In a relationship	235	33.38	145	28.71
	Not in a relationship	376	53.41	360	71.29
	Missing	93	13.21	0	0
	Total	704	100	505	100
Self-reported health	Poor health	191	27.13	115	22.77
	Good health	513	72.87	390	77.23
	Total	704	100	505	100
Discrete/continuous variables					
Income (₵)		922.77	510.46	1407.21	948.01
Education (yrs)		12.12	3.21	18.22	4.32
Age (yrs)		62.91	9.29	61.31	9.95
Frailty		6.23	3.76	21.44	2.27
Sedentary behaviour (mins)		88.74	43.8	1032.36	1086.78

Findings from the t-test on sedentary behaviour: t = -18.10; df = 434.87; *p* < 0.001; 95% CI = ± 204.93; *SD* Standard deviation, *n* Frequency, *%* Percent, n and % apply to categorical variables whereas the mean and SD apply to discrete or continuous variables

Table 3 shows Pearson’s correlation between relevant variables. In the flat neighbourhood, sedentary behaviour was negatively but weakly correlated with frailty (*r* = -0.094; *p* < 0.05; two-tailed) but positively correlated with chronic disease status (*r* = 0.077; *p* < 0.05; two-tailed), which means that sedentary behaviour was lower at higher frailty but was higher among those with at least one chronic condition, compared with those without a chronic condition. In the hilly neighbourhood, sedentary behaviour was positively correlated with frailty (*r* = 0.23; *p* < 0.001; two-tailed) and chronic disease status (*r* = 0.24; *p* < 0.001; two-tailed), which suggests that sedentary behaviour was higher at higher frailty and among those with at least one chronic condition. There was no correlation between sedentary behaviour and poor health in either of the two neighbourhoods (*p* > 0.05).

**Table 3:** Bivariate correlations between sedentary behaviour, health indicators, and covariates in flat and hilly neighbourhoods

Variable	1	2	3	4	5	6	7
Flat neighbourhood (*n* = 704)							
1. Sedentary behaviour	1	− 0.094*	0.077*	-0.034	0.108**	-0.019	-0.048
2. Frailty		1	0.332**	0.671**	0.109**	0.026	0.258**
3. Chronic disease status^a^			1	0.309**	0.056	-0.04	0.423**
4. Poor health^b^				1	0.080*	-0.043	0.214**
5. Women^c^					1	0.011	0.184**
6. Income (₵)						1	0.067
7. Age (yrs)							1
Hilly neighbourhood (*n* = 505)							
1. Sedentary behaviour	1	0.230**	0.236**	0.087	-0.049	-0.026	0.377**
2. Frailty		1	0.150**	0.187**	0.204**	0.022	0.143**
3. Chronic disease status^a^			1	0.360**	0.071	0.007	0.268**
4. Poor health^b^				1	0.144**	0.019	0.152**
5. Women^c^					1	− 0.229**	0.061
6. Income (₵)						1	0.063
7. Age (yrs)							1

^a^ ‘None’ set as a reference group

^b^ ‘Good health’ set as a reference group

^c^ Men set as a reference group

**p < 0.001; *p < 0.05

Table 4 shows regression results relating to the above correlations. In the ultimate model, frailty was negatively associated with sedentary behaviour in the flat neighbourhood (β = -0.18; t = -3.2; *p* < 0.001), which suggests that higher frailty was associated with lower sedentary behaviour. Those with at least one chronic condition reported higher sedentary behaviour in the flat neighbourhood (β = 0.12; t = 2.45; *p* < 0.05), compared with those without a chronic condition. In the hilly neighbourhood, sedentary behaviour was positively associated with frailty (β = 0.16; t = 3.54; *p* < 0.001), which suggests that higher frailty was associated with higher sedentary behaviour. Moreover, those with at least one chronic condition reported higher sedentary behaviour (β = 0.13; t = 2.65; *p* < 0.05), compared with those without any chronic condition. In both neighbourhoods, there was no significant association between poor health and sedentary behaviour (*p* > 0.05).

**Table 4:** The associations of sedentary behaviour with health indicators and covariates between flat and hilly neighbourhoods

Predictor variable	Flat neighbourhood (*n* = 704)				Hilly neighbourhood (*n* = 505)			
	Coefficients				Coefficients			
	B	SE	β(t)	95% CI	B	SE	β(t)	95% CI
Baseline models								
(Constant)	90.567	3.952	(22.92)**	± 15.518	-1213.56	489.596	(-2.479)*	± 1924.585
Frailty	-1.823	0.597	-0.156(-3.05)*	± 2.345	95.037	22.954	0.195(4.14)**	± 90.23
CDS	11.738	3.957	0.118(2.967)*	± 15.536	461.04	110.114	0.212(4.19)**	± 432.855
Poor health	3.382	4.999	0.034(0.676)	± 19.629	-90.347	126.334	-0.036(-0.72)	± 496.615
Age-adjusted models								
(Constant)	111.616	12.528	(8.91)**	± 49.206	-2634.05	510.185	(-5.16)**	± 2005.535
Frailty	-1.477	0.658	-0.122(-2.246)*	± 2.583	69.355	22.155	0.143(3.13)*	± 87.09
CDS	14.228	4.456	0.146(3.193)*	± 17.501	296.683	107.472	0.136(2.76)*	± 422.472
Poor health	-0.891	5.537	-0.009(-0.161)	± 21.748	-87.519	120.159	-0.035(-0.73)	± 472.345
Age (yrs)	-0.365	0.213	-0.077(-1.718)	± 0.835	33.35	4.894	0.314(6.82)**	± 19.238
Ultimate (fully adjusted) models								
(Constant)	84.023	12.846	(6.541)**	± 50.469	-2477.06	519.367	(-4.77)**	± 2041.667
Frailty	-2.082	0.651	-0.182(-3.20)**	± 2.559	78.062	22.044	0.160(3.54)**	± 86.656
CDS^a^	10.71	4.38	0.115(2.45)*	± 17.206	281.867	106.423	0.130(2.65)*	± 418.355
Poor health^b^	-1.67	5.48	-0.017(-0.31)	± 21.529	-38.331	119.997	-0.015(-0.32)	± 471.715
Age (yrs)	0.16	0.22	0.034(0.73)	± 0.864	33.985	5.062	0.320(6.71)**	± 19.901
Women^c^	20.022	3.54	0.235(5.66)**	± 13.976	-337.246	101.973	-0.152(-3.31)**	± 400.865
Income (₵)	0.00	0.003	0.006(0.14)	± 0.013	-0.104	0.052	-0.091(-2.02)*	± 0.203

*SE* Standard error (of B), *CI* Confidence interval (of B), *CDS* Chronic disease status (i.e., one or more conditions), *B* Unstandardised coefficient, *β* Standardised coefficient; sedentary behaviour is the dependent variable in all models; F-test was significant at a minimum of *p* < 0.05 for all models; the total variance explained by predictors in the flat neighbourhood models ranged from 2.1 to 8.2% whereas the variance explained in the hilly neighbourhood models ranged from 9.2 to 20.2%; tolerance ≥ 0.5 for all predictors in the flat and hilly neighbourhood models, and Durbin-Watson values for the models were approximately 2

^a^ ‘None’ set as a reference group

^b^ ‘Good health’ set as a reference group

^c^ ‘Men’ set as a reference group

**p < 0.001; *p < 0.05

In the flat neighbourhood, the regression weight between frailty and sedentary behaviour decreased by 21% from 0.156 (in the baseline model) to 0.122 (in the age-adjusted model). The corresponding change in the hilly neighbourhood was 27%. In the flat neighbourhood model, the regression weight between sedentary behaviour and chronic disease status increased by 24% from 0.118 (in the baseline model) to 0.146 (in the age-adjusted model). In the hilly neighbourhood, the regression weight between sedentary behaviour and chronic disease status decreased by 36% from 0.212 (in the baseline model) to 0.136 (in the age-adjusted model). Thus, between 21% and 36% of the main effect sizes were contributed by age. Even so, age more strongly influenced the associations of sedentary behaviour with frailty and chronic disease status in the hilly neighbourhood.

## Discussion

This study aimed to compare sedentary behaviour and its associations with three health indicators (i.e., frailty, chronic disease status, and poor health) between flat and hilly neighbourhoods. Age and other covariates were adjusted to ascertain whether they influenced the above associations in the two neighbourhoods.

This study found that older adults in the hilly neighbourhood reported higher sedentary behaviour, an outcome signifying a large effect size. This result is analogous to the evidence that hilly neighbourhoods, compared to flatter ones, discourage PA and encourage social isolation involving too much sitting [5–7]. For example, in two qualitative studies [5, 6], older adults attributed their failure to perform PA to the availability of hills in their neighbourhoods. These older adults thought they did not have the functional capacity necessary for sustaining PA in their hilly neighbourhoods. Other studies have similarly reported lower PA for older adults living in hilly areas [7]. Though the level of sedentary behaviour is independent of PA [2, 3], the above pieces of evidence from previous research suggest that older adults are more likely to perform sedentary behaviour due to the availability of hills in their neighbourhood. A few previous studies [12, 13] have suggested that hills in the neighbourhood may encourage PA and consequently buffer sedentary behaviour, but these studies utilised younger samples. Thus, hilly neighbourhoods may support only individuals with the physical functional ability to exercise in hilly terrains.

After adjusting for the ultimate covariates, this study found a negative association between frailty and sedentary behaviour in the flat neighbourhood, which supports the first hypothesis and suggests that older adults with higher frailty reported lower sedentary behaviour in the flatter neighbourhood. In the hilly neighbourhood, higher frailty was associated with higher sedentary behaviour. This difference in how frailty relates to sedentary behaviour in the two neighbourhoods is noteworthy for a couple of reasons. Most previous studies have confirmed a positive correlation between frailty and sedentary behaviour [32–34], but our evidence implies that this relationship can be negative in flatter neighbourhoods where a high physical strength would not be necessary for walking and performing PA. As our evidence relating to the flatter neighbourhood suggests, even frail older adults can perform PA, especially low-intensity PA such as walking. In addition, our evidence suggests the possibility of hilliness confounding the association between frailty and sedentary behaviour, but previous studies [32–34] confirming a positive association between frailty and sedentary behaviour have not considered this potential confounder. Future research examining the effect of frailty on sedentary behaviour should, therefore, incorporate hilliness as a potential covariate.

This study confirmed a positive association between chronic disease status and sedentary behaviour in both neighbourhoods, though this relationship was stronger in the hilly neighbourhood. This evidence confirms the second hypothesis and suggests that older adults with at least one chronic condition, compared with those without any of these conditions, are more likely to report sedentary behaviour. Previous research [14, 20, 35, 36] has reported mixed evidence on this relationship; while some researchers [35] have argued that having at least one chronic condition can motivate people to avoid sedentary behaviour, other researchers [14, 36] have reported chronic disease status as an outcome of sedentary behaviour. Our result suggests that chronic conditions may have underlying physiological problems (e.g., pain and weakness of the body) that can discourage PA. If so, a chronic disease status can be associated with higher sedentary behaviour. We reason further that whether a chronic disease status would be positively associated with sedentary behaviour would depend on age and the type of conditions being faced. The oldest-old (i.e., older adults aged 85 years or higher) or older adults with pain-associated chronic conditions are more likely to avoid PA and perform sedentary behaviour. The hilly neighbourhood, compared to the flatter neighbourhood, possibly presented higher risks of falls in people with relatively low physical functional ability who could not meet high energy requirements for performing PA, which explains why it produced a stronger association between sedentary behaviour and chronic disease status.

Our data did not support the third hypothesis, which is about the association between poor health and sedentary behaviour. Thus, the negative association between sedentary behaviour and poor health was not significant in both neighbourhoods. Previous studies [35, 37–40] have reported mixed findings about this relationship, possibly due to cultural differences between samples and inconsistencies in research design. Thus, while there are studies that have reported a significant association between sedentary behaviour and poor health, the current study and other previous studies do not confirm this association. Our study is unique for assessing this association and providing results on low and hilly neighbourhoods for the first time.

Another unique attribute of this study is the influence of the ultimate covariates in our models. Though age alone had a significant influence on the primary associations tested, it was more influential in the hilly neighbourhood models, suggesting that the associations of frailty and chronic disease status with sedentary behaviour are more dependent on age in the hilly neighbourhood. The result about the role of age in the models is congruent with the argument of the Disengagement Theory of Ageing proposed in the early 1960s [16]. This theory asserts that frailty and other physiological limitations are due to ageing and that frailty and its influence on health and health-seeking behaviours are dependent on age. Our study is significant for supporting this reasoning between the two neighbourhoods.

Worth noting are the associations of sedentary behaviour with chronic disease status. In both neighbourhoods, sedentary behaviour was positively associated with chronic disease status, but the hilly neighbourhood was more strongly associated with chronic disease status. The literature [25, 37] suggests that this stronger association is not necessarily due to the hilliness of the chosen study area. In a study conducted in Ghana [25], living in a rural area was found to be associated with poorer cognitive function, a risk factor for sedentary behaviour. A systematic review has found that more urban neighbourhoods can better support health indicators including PA [37]. Since the hilly neighbourhood was less urban, a stronger association between sedentary behaviour and chronic disease status in it may be due to its semi-urban status rather than its hilliness. Therefore, future studies comparing flat and hilly neighbourhoods with homogeneous and comparable demographics are needed to establish whether the stronger association between sedentary behaviour and chronic disease status in the hilly neighbourhood is due to hilliness.

Our evidence suggests that hilliness may determine whether older adults can avoid social isolation and too much sitting. Smart neighbourhoods (i.e., neighbourhoods that support different types of PA such as walking and bicycling) are known to support individuals to avoid social isolation and sedentary behaviour [41, 42]. By our evidence, flatter neighbourhoods may be smarter than hilly ones with rocky hills for older populations. If so, city planners need to recognise hilliness as a factor that may influence PA and sedentary behaviour in older populations. Specifically, researchers could investigate the role of hilliness in walkability, which is the degree to which a neighbourhood provides resources or attributes (i.e., parks, connected streets, mixed land use, high residential density, services, and safety) that encourage walking and other active forms of transportation [43, 44]. Walkability is a term used to describe a dominant quality of smart cities [43, 45], so future research assessing and confirming the relationship between it and neighbourhood hilliness can demonstrate the role of hilliness in smart cities.

Our results suggest a need for neighbourhoods to be sufficiently flat or sited away from areas with steep hills. City developers should be prepared to invest in levelling hilly areas scheduled to serve as neighbourhoods. Given that some hilly neighbourhoods can encourage PA in younger populations [12, 13], flat neighbourhoods may only be beneficial to older adults with functional limitations. Finally, the burden of diseases associated with sedentary behaviour may be higher in hilly neighbourhoods, given that frailty and the proportion of chronic conditions were higher in the hilly neighbourhood (see Table 2). Hence, more resources may be required to meet the healthcare needs of older adults in hilly communities. Older adults in hilly neighbourhoods need special support or interventions (e.g., designing hilly neighbourhoods in a special way to ease physical activity) to avoid sedentary behaviour. These special interventions may include flattening hilly neighbourhoods and making them more walkable. Since older adults may be unaware of the behavioural health risks posed by hilly neighbourhoods, health education programmes aimed at enabling them to understand and navigate the risks in later life are imperative.

### Strengths and limitations

This study has some limitations. Firstly, as a cross-sectional design, it provides only associations and does not establish causation between the variables. The two neighbourhoods have different demographics (i.e., one being semi-urban and the other being urban), which may have explained the differences in sedentary behaviour and the associations assessed. Even so, this study would at least inform the design of future studies overcoming these limitations. Our sample sizes are relatively small, especially for the hilly neighbourhood. Our sampling method was non-probabilistic and was based on some inclusion criteria, which means that our samples may not be representative of older adults in the two neighbourhoods. For the above reasons, the power of our tests was limited and our findings and result interpretations may have limited generalisability. The sample size of the flat neighbourhood was larger, which implies that the associations from this sample were more likely to be significant. Finally, our data came from self-reported scales, suggesting that they were vulnerable to response bias. We, nevertheless, tried to minimise this type of bias with procedures against CMB. Despite these limitations, this study has several strengths.

This study is novel as it was the first to compare older adults’ sedentary behaviour and its associations with three health indicators between low and hilly neighbourhoods. Similarly, this study was the first that focused on an African sample. It also employed a robust statistical analysis in which covariates including age were adjusted for, enabling researchers and decision-makers to understand how age may differently affect the associations of the health indicators with sedentary behaviour in the two neighbourhoods. This study followed the STROBE, which means that it met all relevant quality indicators of the cross-sectional design [22, 46]. Additional file 5 shows the STROBE checklist items met. Our sensitivity analyses enabled us to minimise confounding and assess the influence of the covariates on the primary associations assessed. Without these analyses, this study would have reported wrong regression weights and statistical significance. Finally, our robust cross-sectional design can serve as a model for future research.

## Conclusions

Sedentary behaviour was higher in the hilly neighbourhood, and older adults with at least one chronic condition reported higher sedentary behaviour in both neighbourhoods, though those in the hilly neighbourhood reported more sedentary behaviour. Older adults in the flat neighbourhood reported lower sedentary behaviour at higher frailty, but their counterparts in the hilly neighbourhood reported higher sedentary behaviour at higher frailty. The associations of frailty and chronic disease status with sedentary behaviour depended much on age, especially in the hilly neighbourhood. Older adults with higher frailty and a chronic disease status in the hilly neighbourhood were more likely to report sedentary behaviour. This study implies that older adults in hilly neighbourhoods need special support or interventions (e.g., designing hilly neighbourhoods in a special way to ease physical activity) to avoid sedentary behaviour. Chronic conditions associated with sedentary behaviour may be more prevalent in hilly neighbourhoods, which suggests that the burden of these conditions may be higher in hilly communities. As such, higher healthcare expenditures may be needed to meet the needs of older adults with chronic conditions in hilly neighbourhoods.

### Supplementary Information

**Additional file 1.** Items used measure sedentary behaviour.

**Additional file 2.** The scale used to measure frailty.

**Additional file 3.** Assumptions assessed for the independent samples t-test and hierarchical linear regression.

**Additional file 4.** Steps taken in the first sensitivity analyses for confounding variables.

**Additional file 5.** STROBE Statement—checklist of items met.

**Additional file 6.** a. Lowland_Darkuman_Data. b. Highland_Abetiffi_Data.

## Data Availability

Data used for this study are available as an online supplementary material (i.e., Additional file 6a and b).
