# Supplementary material for: Sedentary behaviour among older adults residing in flat and hilly neighbourhoods and its association with frailty and chronic disease status
Source: BMC Public Health. 2023 Oct 24;23:2083. doi: 10.1186/s12889-023-17029-0 (PMC10599026; doi:10.1186/s12889-023-17029-0)
Supplement: Supplementary file 1 — Additional file 1. Items used measure sedentary behaviour. [file 12889_2023_17029_MOESM1_ESM.doc]

Additional file 1. Items used measure sedentary behaviour

The following items describe your sitting habits. In the last 7 days, how much **time (in minutes)** did you spend on each habit on a typical day? Please insert **0** or **NIL** if you didn’t perform the habit.

| No. | Habit | Time (minutes) |
| --- | --- | --- |
| 1 | Sitting in a car as a passenger |  |
| 2 | Sitting while viewing the television |  |
| 3 | Listening to radio or any form of audio (e.g., music) when sitting or lying down |  |
| 4 | Sitting while reading (a book, newspaper, magazine) |  |
| 5 | Sitting idle or reclining |  |
| 6 | Lying down to rest (but keeping awake) |  |
