# Supplementary material for: Sedentary behaviour among older adults residing in flat and hilly neighbourhoods and its association with frailty and chronic disease status
Source: BMC Public Health. 2023 Oct 24;23:2083. doi: 10.1186/s12889-023-17029-0 (PMC10599026; doi:10.1186/s12889-023-17029-0)
Supplement: Supplementary file 2 — Additional file 2. The scale used to measure frailty. [file 12889_2023_17029_MOESM2_ESM.doc]

Additional file 2. The scale used to measure frailty

| No. | Item | Response | |
| --- | --- | --- | --- |
|  |  | No | Yes |
| 1 | Do you feel physically healthy? |  |  |
| 2 | Have you lost a lot of weight recently without wishing to do so? |  |  |
| 3 | Do you experience problems in your daily life due to difficulty in walking? |  |  |
| 4 | Do you experience problems in your daily life due to difficulty maintaining your balance? |  |  |
| 5 | Do you experience problems in your daily life due to poor hearing? |  |  |
| 6 | Do you experience problems in your daily life due to poor vision? |  |  |
| 7 | Do you experience problems in your daily life due to lack of strength in your hands? |  |  |
| 8 | Do you experience problems in your daily life due to physical tiredness? |  |  |
| 9 | Do you have problems with your memory? |  |  |
| 10 | Have you felt down during the last month? |  |  |
| 11 | Have you felt nervous or anxious during the last month? |  |  |
| 12 | Are you able to cope with problems well? |  |  |
| 13 | Do you live alone? |  |  |
| 14 | Do you sometimes miss having people around you? |  |  |
| 15 | Do you receive enough support from other people? |  |  |

**Coding**: No – 0; Yes – 1; 5 is the base cut-off score of frailty on the scale

Source: Dong et al. (2017)
