# Supplementary material for: Sedentary behaviour among older adults residing in flat and hilly neighbourhoods and its association with frailty and chronic disease status
Source: BMC Public Health. 2023 Oct 24;23:2083. doi: 10.1186/s12889-023-17029-0 (PMC10599026; doi:10.1186/s12889-023-17029-0)
Supplement: Supplementary file 3 — Additional file 3. Assumptions assessed for the independent samples t-test and hierarchical linear regression. [file 12889_2023_17029_MOESM3_ESM.doc]

Additional file 3. Assumptions assessed for the independent samples t-test and hierarchical linear regression

| # | Assumption | Step | Result | Decision |
| --- | --- | --- | --- | --- |
| 1 | Homogeneity of variances of data from the two neighbourhoods | The Levene’s test of equality of variances was performed as part of the independent samples t-test | The test was significant, which evidenced a violation of the assumption. | Since the assumption was violated, we interpreted and used results corresponding to “equal variances not assumed” (Garson, 2012). |
| 2 | Normality of the data associated with the dependent variable | Assessed normality with the Shapiro-Wilk’s test of normality | The Shapiro-Wilk’s test produced a non-significant result at p >0.05 for the low-altitude neighbourhood but not for the other neighbourhood. | We analysed the data despite a violation of the assumption for one of the neighbourhoods because our sample size was relatively large (Garson, 2012). |
| 3 | Linearity of the associations | We plotted standardized residuals against standardized predicted values of the dependent variable in HLR analysis models fitted. | The graph shows a linear cluster of values and a straight line as recommended (Garson, 2012) | Assumption or condition was met |
| 4 | Independence of regression errors | Durbin Watson statistics were generated for all the HLR models fitted. | Durbin-Watson statistic was approximately 2 as recommended (Garson, 2012) | The assumption was met |
| 5 | Multi-collinearity | Tolerance values were computed through the HLR models. | The tolerance values are >0.2 as recommended (Garson, 2012) | The assumption was met |
| 6 | Homogeneity of variances around the regression line | We plotted standardized residuals against standardized predicted values of the dependent variable in the HLR models fitted. | The graphs produced a satisfactory pattern as recommended (Garson, 2012) | The assumption was met |

**Note**: HLR – hierarchical linear regression
