# Supplementary material for: Sedentary behaviour among older adults residing in flat and hilly neighbourhoods and its association with frailty and chronic disease status
Source: BMC Public Health. 2023 Oct 24;23:2083. doi: 10.1186/s12889-023-17029-0 (PMC10599026; doi:10.1186/s12889-023-17029-0)
Supplement: Supplementary file 4 — Additional file 4. Steps taken in the first sensitivity analyses for confounding variables. [file 12889_2023_17029_MOESM4_ESM.doc]

Additional file 4. Steps taken in the first sensitivity analyses for confounding variables

| Stage | # | Action |
| --- | --- | --- |
| 1 | 1 | Fit a simple linear regression model to assess the relationship between frailty and sedentary behaviour |
|  | 2 | Note the standardised regression weight from step 1 |
|  | 3 | Fit a multiple linear regression model in which all measured confounding variables are treated as predictors of the main independent variable, sedentary behaviour |
|  | 4 | Identify from step 3 potential confounders that have a p-value ≥0.25 |
|  | 5 | Predictors from step 4 that produced a p≥0.25 should be removed from the analysis and the others kept for the next stage of the analysis |
| 2 | 6 | Adjust for each of the remaining confounding variables in the model fitted at step 1 |
|  | 7 | Compute the per cent change between the standardised regression weight at step 1 and the new weight resulting from step 6 |
|  | 8 | All potential confounders that produce a change of 10% or more should be incorporated into the final analysis as the ultimate confounders |
|  | 9 | Repeat the process (i.e., steps 1-8) for each of the other predictors (i.e., poor health and chronic disease status) |

**Note**: The ultimate confounders were variables retained in the process across the three predictors. Income, age, and gender were the ultimate confounders found.
